# Optimizing staging of Meniere’s disease: integrating electrocochleography with vestibular tests

**DOI:** 10.3389/fnins.2025.1600665

**Published:** 2025-07-08

**Authors:** Muchen Huang, Xianren Wang, Minqi Chen

**Affiliations:** ^1^Department of Otorhinolaryngology, The First Affiliated Hospital, Sun Yat-sen University, Guangzhou, China; ^2^Institute of Otorhinolaryngology, Sun Yat-sen University, Guangzhou, China; ^3^Department of Otorhinolaryngology, The Second People’s Hospital of Foshan, Foshan, China

**Keywords:** Meniere’s disease, electrocochleography, vestibular function tests, diagnostic staging, inner ear dysfunction

## Abstract

**Objectives:**

This study aimed to evaluate the diagnostic and staging efficacy of integrating electrocochleography (ECochG) with vestibular function tests—specifically cervical and ocular vestibular evoked myogenic potentials (cVEMP and oVEMP), caloric test (CT), and video head impulse test (vHIT)—for Ménière’s disease (MD).

**Design:**

Data were collected from 54 MD patients (66 affected ears) admitted to a hospital between January 2023 and January 2024. Each participant underwent pure tone audiometry, ECochG, cVEMP, oVEMP, CT, and vHIT. The results were compared against both established clinical criteria and a newly proposed staging system. Inclusion criteria followed the 2015 diagnostic guidelines for MD. Statistical analyses, including ANOVA, Chi-square, and Kruskal–Wallis H tests, were conducted, and a random forest model was employed to validate the robustness of the proposed staging system.

**Results:**

The novel staging system, incorporating vestibular function tests, demonstrated superior sensitivity and diagnostic accuracy compared to traditional audiometry-based staging. Early-stage MD detection improved significantly, with vestibular test abnormalities strongly correlating with disease progression. The overall positive rate for any test was 98.5%. ECochG abnormalities were detected in 54.5% of cases, while cVEMP and oVEMP abnormalities were observed in 75.8% and 69.7% of cases, respectively. The new staging system exhibited a stronger correlation with vestibular dysfunction, effectively identifying functional impairment prior to significant hearing loss.

**Conclusion:**

The integration of ECochG with vestibular function tests provides a more comprehensive diagnostic framework for MD. This multimodal approach enhances early detection, improves staging accuracy, and offers deeper insights into disease progression, thereby facilitating more personalized treatment strategies.

## Highlights

• Developed a novel staging system for Meniere’s disease integrating cochlear and vestibular tests.
• Demonstrated superior sensitivity in early-stage detection compared to PTA-based staging.
• A multimodal approach could better understand and treat inner ear dysfunction.
• Emphasized the importance of standardized vestibular testing and personalized interventions for severe cases.

## 1 Introduction

Ménière’s disease (MD) is a disorder characterized by recurrent episodes of vertigo, hearing loss, tinnitus, and aural fullness. The etiology of MD is complex and multifactorial, with several mechanisms potentially contributing to its onset. Genetic susceptibility, including rare mutations in both familial and sporadic cases, has been implicated, particularly in genes like FAM136A, which affect endolymphatic metabolism (Frejo et al., 2025; Frejo et al., 2017). Immune dysregulation also plays a key role, with evidence of persistent systemic inflammation driven by both proinflammatory cytokines and allergic responses, especially involving type 2 cytokines such as IL-4, IL-5, and IL-13. Furthermore, autoinflammatory processes involving IL-1β and the NLRP3 inflammasome have been shown to contribute to the disease’s pathogenesis (Frejo et al., 2017; Flook et al., 2024). These factors collectively suggest that MD arises from a combination of genetic and immune-mediated mechanisms, adding to the complexity of its etiology and treatment. Histopathological studies have linked it to endolymphatic hydrops (ELH), an abnormal accumulation of endolymph fluid in the membranous labyrinth of the inner ear (Mohseni-Dargah et al., 2023).

While MD is generally considered incurable, early diagnosis and timely intervention are crucial for mitigating its debilitating symptoms. The 1995 diagnostic criteria established by the American Academy of Otolaryngology-Head and Neck Surgery classify MD staging based on pure-tone audiometry (PTA). The staging system considers the arithmetic mean of pure-tone thresholds at 0.5, 1, 2, and 3 kHz from the worst audiogram within 6 months prior to treatment. Stages are defined as follows: stage I (<26 dB), stage II (26–40 dB), stage III (41–70 dB), and stage IV (>70 dB) (Sun et al., 2021). For bilateral MD, each ear is staged independently. While PTA remains the primary method for assessing hearing loss (Jalali et al., 2022), it often fails to capture the asynchronous onset of cochlear and vestibular symptoms, which may emerge months or even years apart (Xu et al., 2024).

Endolymphatic hydrops, the hallmark pathology of MD, affects both the cochlear and vestibular systems, including the semicircular canals, saccule, and utricle (van Steekelenburg et al., 2020). Consequently, a comprehensive evaluation of vestibular function is essential for accurate staging. Vestibular function can be assessed using a battery of tests, including electrocochleography (ECochG), cervical vestibular evoked myogenic potential (cVEMP), ocular vestibular evoked myogenic potential (oVEMP), caloric test (CT), and video head impulse test (vHIT). Each test targets specific structures of the inner ear, providing a complete overview of both cochlear and vestibular involvement (Kunelskaya et al., 2023).

Electrocochleography measures the cochlea’s electrical responses and auditory nerve activity. It includes three components: cochlear microphonic potential (CM), summating potential (SP), and auditory nerve action potential (AP). The CM and SP reflect outer hair cell function, while the AP corresponds to wave I of the auditory brainstem response (ABR). The SP/AP ratio, with a threshold of >0.4, is considered abnormal and has a sensitivity of 50%–70% for detecting ELH (Hornibrook, 2023).

Vestibular evoked myogenic potentials (VEMPs) are tests used to assess vestibular function. The cVEMP provides an ipsilateral, inhibitory response, indicating saccular and inferior vestibular nerve function. In contrast, the oVEMP provides a crossed, excitatory response, reflecting the functioning of the utricle and the superior vestibular nerve pathway. Together, they offer a noninvasive and specific assessment of otolith organ function (Colebatch et al., 2016). Additionally, the CT evaluates low-frequency function of the horizontal semicircular canals, while the vHIT examines the high-frequency function of all six semicircular canals and their respective vestibular pathways via vestibular-ocular reflex (VOR) (Alhabib and Saliba, 2022; Tamanini et al., 2023; Waissbluth et al., 2022).

Currently no gold standard for diagnosing MD, particularly in its early stages when symptoms are atypical. Clinicians often rely on the patient’s medical history, auxiliary examinations, and their response to drug treatment to reach a diagnosis (Güneri et al., 2016). This study aims to evaluate the diagnostic utility of combining ECochG with vestibular function tests and proposes a novel staging system. The findings may provide a more comprehensive foundation for diagnosing, managing, and prognosticating MD.

## 2 Materials and methods

### 2.1 Clinical data

Between January 2023 and January 2024, 54 patients with MD were admitted to the First Affiliated Hospital of Sun Yat-sen University. This cohort comprised 42 patients with unilateral MD and 12 patients with bilateral MD, resulting in a total of 66 affected ears. Among these, 25 ears were from male patients (22 cases), and 41 ears were from female patients (32 cases). The average age of the patients was 55 years, with a range of 15–79 years.

### 2.2 Inclusion/exclusion criteria

The inclusion criteria were as follows: Patients were diagnosed with definite MD according to the 2015 diagnostic criteria established by the American Academy of Otolaryngology-Head and Neck Surgery, the Barány Society, and the European Academy of Otology and Neurotology (Basura et al., 2020). These criteria included: (1) two or more spontaneous episodes of vertigo lasting between 20 min and 12 h; (2) audiometrically documented low- to medium-frequency sensorineural hearing loss in one ear, with the affected ear identified before, during, or after at least one episode of vertigo; and (3) fluctuating aural symptoms in the affected ear, such as changes in hearing, tinnitus, or a sensation of fullness. Additionally, these symptoms could not be better explained by another vestibular diagnosis.

The exclusion criteria were: (1) age under 18 years; (2) a history of prior temporal bone surgery or cochlear implant placement; (3) inability to provide informed consent; (4) severe disability (e.g., neurological, orthopedic, or cardiovascular) or serious concurrent illness that might interfere with treatment or follow-up; and (5) active neuro-otologic disorders that may mimic MD, such as vestibular migraine, recurrent vestibulopathy, phobic postural vertigo, vertebrobasilar transient ischemic attacks (TIAs), or acoustic neuroma.

### 2.3 Staging criteria

All patients underwent PTA, ECochG, cVEMP and oVEMP, CT, and vHIT. Traditional clinical staging was based on PTA thresholds, while the new staging system incorporated abnormalities detected across the five tests. The new system categorized stages as follows: stage I (≤2/5 abnormalities), stage II (3/5 abnormalities), stage III (4/5 abnormalities), and stage IV (5/5 abnormalities).

### 2.4 Test protocols

#### 2.4.1 Pure-tone audiometry

Air- and bone-conduction thresholds were measured in ISO 8253-1 compliant soundproof chambers using a Madsen Conera clinical audiometer (GN Otometrics, Denmark). The test protocol encompassed 10 frequencies (125, 250, 500, 1,000, 1,500, 2,000, 3,000, 4,000, 6,000, and 8,000 Hz) with 5 dB intensity increments following a modified Hughson-Westlake procedure. Masking was systematically applied using narrowband noise when interaural differences exceeded 40 dB HL for air conduction or 15 dB HL for bone conduction, thereby ensuring ear-specific threshold validity. The pure-tone average (PTA) was calculated as the mean threshold at four core speech frequencies (500, 1,000, 2,000, and 4,000 Hz) in accordance with AAO-HNS guidelines for MD staging.

#### 2.4.2 Electrocochleography

Subjects were positioned in lateral decubitus within a soundproof chamber following otoscopic confirmation of intact tympanic membranes and patent external auditory canals. After alcohol-based canal cleaning, a saline-soaked tympanic membrane electrode (Tiptrode^®^) was placed at the posteroinferior quadrant under direct visualization, with reference and ground electrodes positioned on the ipsilateral earlobe and forehead, respectively. Alternating polarity clicks (90 dB nHL, 7.1 Hz repetition rate) were delivered via insert earphones, and bioelectric signals were recorded using the Intelligent Hearing Systems with a 10–1,500 Hz bandpass filter and 100 signal averages. The analysis window spanned −5 to 15 ms post-stimulus. Diagnostic thresholds for ELH followed established criteria, defined as an SP/AP amplitude ratio >0.4, with automated artifact rejection excluding waveforms exceeding ±20 μV (Eggermont and Odenthal, 1974).

#### 2.4.3 Vestibular evoked myogenic potentials

##### 2.4.3.1 Cervical vestibular evoked myogenic potential

The subject sat upright and turned his head to the opposite side as much as possible to maintain the tension of the sternocleidomastoid muscle (real-time monitoring of myoelectric activity 20–40 μV). The recording electrode was placed on the upper 1/3 of the sternocleidomastoid muscle, the reference electrode was on the ipsilateral sternocleidomastoid joint, and the ground pole was on the forehead. A 500 Hz short pure tone (rise/fall 1 ms, plateau 2 ms) was used, the intensity was 95 dB nHL, the stimulation rate was 5 Hz, and it was superimposed 100 times. The filter bandwidth was 50–1,500 Hz, and the analysis window was 0–60 ms. The bilateral asymmetry ratio (AR) was calculated based on the P13-N23 amplitude, and the formula was: AR = (|A right − A left|)/(A right + A left) × 100%, and AR > 34% was abnormal.

##### 2.4.3.2 Ocular vestibular evoked myogenic potential

The subject lay supine and maintained an upward gaze of 30°. The recording electrode was placed 1 cm below the lower eyelid, the reference electrode was the mandibular symphysis, and the ground pole was on the forehead. A total of 500 Hz bone conduction vibration (B71 vibrator, RION) was used, with an intensity of 130 dB FL, a stimulation rate of 5 Hz, and 150 superpositions. The filter bandwidth was 10–1,000 Hz, and the analysis window was 0–50 ms. N10-P15 amplitude < 4 μV or AR > 35% was considered abnormal (Rosengren et al., 2019).

#### 2.4.4 Caloric test

The Otoaccess nystagmus analysis system was used. The patients were placed in a supine position with their heads tilted forward 30°. 30°C cold water and 44°C warm water (non-perforated ears) or 24°C cold air/50°C hot air (perforated ears) were perfused in a random order for 30 s on each side with an interval of 5 min. The slow phase velocity (SPV) was recorded 60–70 s after perfusion, and the unilateral attenuation value (UW) was calculated. UW > 25% was considered abnormal. During the perfusion, the patients were required to remain alert by mental arithmetic, and the fixation of the light spot was recorded for 10 s during the fixation suppression phase (Norré, 1987).

#### 2.4.5 Video head impulse test

The ICS Impulse system was used. The patient sat and fixed his gaze on a 1 m target. The examiner applied a 10°–20° instantaneous head movement (peak velocity 150–300°/s) in the horizontal/vertical semicircular canal plane. The normal range of gain for the horizontal semicircular canal was 0.8–1.2, and for the vertical canal was 0.7–1.2. A unilateral gain reduction of >0.1 accompanied by corrective saccades (≥3 times) was considered abnormal. Each semicircular canal was tested 20 times, and the system automatically eliminated invalid trials with insufficient head velocity (<100°/s) or following eye movements (gain > 2.0) (Zamaro et al., 2020).

### 2.5 Statistical analysis

Data were analyzed using SPSS version 29.0. ANOVA and the Kruskal–Wallis H test were used to compare continuous and ordered categorical data, respectively. Correlation analyses were carried out using Kendall’s tau-c. A *P*-value < 0.05 was considered statistically significant. The new staging system validation employed a Random Forest Classifier configured with 100 decision trees using Gini impurity minimization for node splitting. The model architecture specified no depth restriction (max_depth = None), required a minimum of 2 samples for node splitting (min_samples_split = 2), and 1 sample per leaf node (min_samples_leaf = 1). Feature selection at each split automatically adopted the square root of total features (max_features = “auto”), with bootstrap sampling enabling out-of-bag (OOB) validation. The complete dataset (*N* = 66) was partitioned into stratified training (70%, *n* = 46) and testing (30%, *n* = 20) subsets, preserving class distribution across both sets.

## 3 Results

A total of 54 patients with MD were analyzed, comprising 42 unilateral cases and 12 bilateral cases, resulting in 66 affected ears. The cohort included 22 males (25 affected ears) and 32 females (41 affected ears), with a mean age of 53.50 years (±12.21), ranging from 15 to 79 years. Among the affected ears, 38 were left-sided, and 28 were right-sided. The average hearing threshold was 57.82 dB HL (±22.73) in affected ears and 36.48 dB HL (±27.05) in contralateral ears.

A summary of clinical characteristics and comorbidities is presented in Table 1. The mean age of onset was 47.03 years (±11.65), and the average disease duration was 6.47 years (±6.58). Migraine was present in 9.26% of patients, while 29.63% had hypertension, 16.67% had diabetes, and 48.15% had high cholesterol. Other comorbidities included sleep disorders (24.07%), anxiety/depression (11.11%), and a family history of MD or related disorders (3.70%). Additionally, 7.41% of patients had a history of ear surgery, including intratympanic injection of gentamicin or endolymphatic sac decompression.

**TABLE 1:** Clinical characteristics and comorbidities of Meniere’s disease patients.

Variables	Statistics (N = 54)	Remark
**Demographics**		
Age	53.50 ± 12.21	
Gender		
Male	22 (40.74%)	
Female	32 (59.26%)	
**Disease characteristics**		
Age of onset	47.03 ± 11.65	
Course of disease	6.47 ± 6.58	
Unilateral/bilateral	42 (77.78%)/12 (22.22%)	
**Comorbidities**		
Hypertension	16 (29.63%)	
Diabetes	9 (16.67%)	
High cholesterol	26 (48.15%)	
Sleep disorders	13 (24.07%)	
Anxiety/depression	6 (11.11%)	
Migraine	5 (9.26%)	Diagnosis according to ICHD-3 criteria
**Family history**		
MD or related disorders	2 (3.70%)	Immediate relatives with MD
**Others**		
History of ear surgery	4 (7.41%)	Intratympanic injection of gentamicin; endolymphatic sac decompression

**FIGURE 1:**
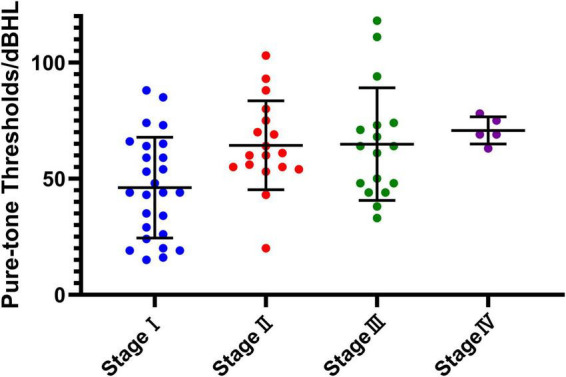
The average hearing threshold distribution at each new stage. The horizontal axis represents the four stages (stage I to stage IV) of the new classification system, while the vertical axis shows the pure-tone hearing thresholds measured in decibels hearing level (dBHL). Individual data points represent measurements for each subject within the corresponding stage, with the central horizontal line indicating the mean value and the error bars representing the standard deviation (SD). This figure illustrates the variation in hearing thresholds and the progression across stages.

Based on clinical staging, the distribution of affected ears was as follows: stage I (10.6%), stage II (9.1%), stage III (54.5%), and stage IV (25.8%). Using the newly proposed staging system, the distribution shifted to stage I (39.4%), stage II (27.3%), stage III (25.8%), and stage IV (7.6%). This reclassification highlights the improved sensitivity of the new staging system, particularly in identifying early-stage MD. The average hearing threshold distribution is illustrated in Figure 1.

The overall positive detection rate across the five diagnostic tests—ECochG, cVEMP, oVEMP, CT, and vHIT—was 98.5%, with 65 out of 66 tests yielding positive results. Individually, the detection rates were as follows: ECochG (54.5%), cVEMP (75.8%), oVEMP (69.7%), CT (66.7%), and vHIT (24.2%).

Statistical analysis revealed a significant correlation between clinical stage and gender (*P* = 0.025), with females exhibiting a higher proportion of severe stages (III and IV). However, no significant correlation was observed between age and clinical stage (*P* = 0.818) or between age and the new staging system (*P* = 0.236). Gender did not significantly influence the new staging system (*P* = 0.756), demonstrating its robustness across demographic variables.

The Kruskal–Wallis H test indicated significant differences in diagnostic test results across stages for ECochG (*P* < 0.001), cVEMP (*P* < 0.001), oVEMP (*P* = 0.003), CT (*P* = 0.004), and vHIT (*P* < 0.001). Pairwise comparisons revealed significant differences between stage I and stages III and IV (*P* < 0.05), reflecting increased test abnormalities with disease progression. However, differences between stage I and stage II were insignificant, suggesting relatively stable abnormality rates in early stages.

**FIGURE 2:**
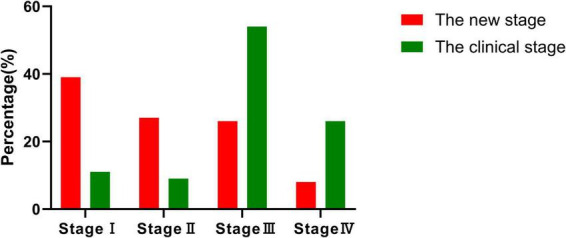
Comparison of staging distribution. The figure illustrates the distribution of 66 patients across four stages (stages I–IV) using both the new and clinical staging methods. Notably, most patients in the new staging system are classified into stage I and stage II, highlighting its superior ability to facilitate early-stage diagnosis compared to the clinical staging method.

A weak positive correlation was observed between the clinical and new staging systems (τ = 0.260, *P* = 0.003). Clinical staging heavily emphasized hearing loss, with over half of the cases (54.5%) classified as stage III. In contrast, the new system identified a larger proportion of early-stage cases—stage I (39.4%) and stage II (27.3%)—better reflecting functional impairment of the cochlea and vestibule (Table 2 and Figure 2).

**TABLE 2:** Distribution of patients with MD in the clinical stage and new stage.

		The clinical stage				
		Stage I	Stage II	Stage III	Stage IV	Total
The new stage	Stage I	6	4	12	4	26
	Stage II	1	0	12	5	18
	Stage III	0	2	9	6	17
	Stage IV	0	0	3	2	5
	Total	7	6	36	17	66

Random forest modeling validated the robustness of the new staging system, achieving an accuracy of 90%, a precision of 91.63%, a recall of 90%, and an F1-score of 0.90. Among the diagnostic tests, ECochG (22.51%) and CT (21.22%) contributed most significantly to the model’s predictive accuracy, followed by vHIT (20.68%), oVEMP (20.43%), and cVEMP (15.16%).

The new staging method holds significant value for the early diagnosis of MD and shows potential for prognostic assessment. By comprehensively evaluating changes in vestibular and auditory function, this method aids in identifying early damage and provides insights into disease progression. Current research focuses on applying this method to guide personalized treatment and predict disease outcomes. We believe this staging system will enhance diagnostic accuracy, provide clinicians with more precise monitoring tools, and ultimately improve patients’ quality of life through more effective treatment strategies.

## 4 Discussion

In this study, according to the clinical hearing loss classification, 10.6% (7/66) of the patients with MD were in stage I, 9.1% (6/66) in stage II, 54.5% (36/66) in stage III, and 25.8% (17/66) in stage IV, with stage III being the most common, which is consistent with the proportion of clinical stages of patients with MD in previous data (Huang et al., 2011; Huang et al., 2020). Among the ears affected, a male-to-female ratio of 1:1.45 indicates a significantly higher prevalence in females. The average age of the patients was 54.1 years (±11.9), with an age of onset ranging from 15 to 79 years. These findings are consistent with the sex ratio and mean age reported in prior studies. Additionally, this study revealed a positive rate of 98.5% (65 out of 66) across any of the five tests conducted. This high sensitivity suggests that combining ECochG with vestibular function tests enhances the assessment of cochlear and vestibular functions in MD patients during clinical practice (Huang et al., 2020).

One of the most important findings of this research is that the novel system can detect early-stage MD. The new system classified a greater proportion of patients as stage I or II compared to traditional PTA staging, demonstrating its sensitivity in identifying vestibular and cochlear dysfunction before significant hearing loss occurs. This early detection is vital for implementing timely interventions that can potentially slow disease progression and improve long-term outcomes.

Sun et al. proposed a staging method based on a combination of vestibular function tests in their study (Sun et al., 2021). This method systematically evaluates the functional status of each component of the vestibular system (including the semicircular canals, utricle, and saccule) by integrating multiple examination methods such as CT and VEMP. This multimodal vestibular function assessment method can more comprehensively reflect the scope of damage to the vestibular system. Evaluating the extent of vestibular involvement is limited, particularly for conditions that impact both the cochlea and the vestibule, such as sudden deafness accompanied by vertigo or MD. A single assessment of vestibular function may not accurately represent the overall severity of the underlying pathophysiological mechanisms of these diseases.

At present, the staging model based on PTA is widely used in clinical practice. This model mainly reflects the severity of the disease by evaluating the degree of damage to cochlear hair cells. As a classic audiometric method, PTA plays an important role in the diagnosis and staging of MD. However, PTA mainly targets cochlear function and cannot directly assess damage to the vestibular system, which limits its application in the comprehensive assessment of otological diseases to some extent. In this study, we proposed a new cochlear plus vestibular staging method, which comprehensively evaluates the involvement of the cochlear and vestibular systems by combining the results of PTA and vestibular function examinations. The results showed that the new staging method was significantly correlated with the current clinical staging (based on PTA) (*P* < 0.05), indicating that the new method has comparable efficacy to PTA in assessing disease severity. However, the correlation coefficient between the two was low(τ = 0.260,*P*=0.003), which may be related to the following factors: first, PTA and vestibular function examination target the cochlea and vestibular systems, respectively, and there are differences in the target organs and pathological mechanisms they evaluate (Kim et al., 2019); second, the vestibular system has a strong compensatory ability, especially in chronic vestibular diseases, the compensation of vestibular function may mask the actual degree of damage; in addition, individual differences (such as age, disease course, and etiology) may also affect the correlation between the two staging methods.

The most typical pathological change of MD is ELH, and an increased SP/AP ratio of ECochG is a diagnostic indicator of ELH (Melki et al., 2014). The sensitivity of the SP/AP ratio is approximately 50%–70%, which aligns with the study’s finding of a 54.5% positive rate for ECochG abnormalities. It is generally accepted that ELH leads to the expansion of the basement membrane due to edema, which affects the tympanic scale. Asymmetric vibration of the basement membrane causes an enlargement of the SP, resulting in an elevated SP/AP ratio (Ishiyama et al., 2017). A ratio greater than 0.4 is considered abnormal (Ge and Shea, 2002). Although MD is typically associated with the presence of EH, the SP/AP ratio does not always show an abnormal increase. This discrepancy may occur because damage to the auditory nerve and hair cells can occur during the progression of MD to irreversible sensorineural hearing loss (Lamounier et al., 2017). When this happens, the endolymphatic pressure may not be elevated, vertigo may subside, and the potentials measured by ECochG can be reduced or undetectable, leading to a lower SP/AP ratio in the later stages of the disease (van Tilburg and Rauch, 2014).

There are various theories regarding the etiology of ELH. Disturbances in microcirculation within the cochlea and imbalances in the production and absorption of endolymphatic fluid can lead to fluid accumulation in the membranous labyrinth (Teggi et al., 2019). Initially, the lower part of the labyrinth (saccule and cochlea) is affected in the development of hydrops, while the upper part (utricle and semicircular canals) may be involved later, albeit less frequently. These changes may eventually lead to vertigo, tinnitus, and hearing loss (Silva et al., 2017). Therefore, a combination of tests to evaluate vestibular organ function is necessary.

Cervical and ocular vestibular evoked myogenic potentials are used to assess the function of the saccule-inferior vestibular nerve and utricle-superior vestibular nerve pathways in MD patients, respectively (Taylor et al., 2016). Given the order of involvement in EH, MD primarily affects cVEMP, followed by oVEMP. Currently, most research on MD focuses on cVEMP. cVEMP is an inhibitory bulbar cervical muscle reflex test that can show characteristic changes in the symptomatic ears of MD patients and may detect early bulbar hydrops before the onset of typical MD symptoms, which is of great significance for the early diagnosis of MD (Young, 2013). Previous studies have demonstrated that cVEMP can accurately identify the affected side in 80% of MD patients. This study’s 75.8% detection rate aligns closely with these findings. Additionally, the difference in binaural cVEMP amplitude is significantly related to the stage of MD, aiding in assessing the disease’s severity and the potential for bilateral involvement. This method is valuable for predicting bilateral lesions (Noij et al., 2019). The VEMP examination method is clinically significant in MD, and abnormal cVEMP can predict hearing loss in mild MD (Murofushi et al., 2016). In this study, the non-elicitation rate and abnormal rate of VEMP in ears from stage I to stage IV were correlated with the average pure tone audiometry threshold (Cheng et al., 2022). This suggests that as the clinical stage progresses, reflecting worsening hearing loss, the function of the saccule and utricle gradually diminishes. There was also a correlation between the results of cVEMP and oVEMP, indicating that both tests correspond to the synchronous progression of the disease, aligning with the progression of ELH.

Both the CT and vHIT assess the function of the semicircular canals, but they measure different frequency ranges. The CT evaluates the ultra-low frequency range of 0.003–0.004 Hz, while the vHIT examines the high-frequency range of 2–5 Hz (Limviriyakul et al., 2020). The abnormal detection rates for these tests are 66.7% and 24.2%, respectively. This suggests that the ultra-low frequency area of the semicircular canals is more vulnerable to damage than the high-frequency area, and the severity of this damage is greater. MD first shows a decrease in the low-frequency function of the semicircular canals, and then high-frequency damage occurs. One possible explanation for this pattern is that the central adaptation mechanism is more readily triggered by physiological stimulation, such as stimuli encountered in daily life. The CT involves non-physiological vestibular stimulation, whereas the vHIT provides physiological stimulation that aligns closely with typical daily activities, making it easier for the system to compensate for (Lee et al., 2017). Given these insights, it appears that vHIT’s contribution to the early diagnosis of MD may be limited. Future research could consider excluding this aspect to improve the effectiveness of staging models. Additionally, expanding the sample size could allow for comparing vHIT results in the early and late stages of MD, helping to evaluate its significance for late-stage disease assessment (Schubert et al., 2004).

After validation, the new staging model demonstrates improved robustness and reliability. It also provides a more balanced distribution of individuals across different genders and age groups at various stages of MD, eliminating the need for adjustments based on age and gender. It is important to note that hearing loss often worsens with age, and female patients with MD tend to experience more severe hearing loss than their male counterparts. Additionally, fluctuations in female hormone levels can significantly affect the health of the auditory system. The current MD staging based on PTA cannot fully account for the interference caused by these confounding factors. Therefore, in clinical diagnosis and treatment, it is crucial to closely monitor and intervene in cases of hearing fluctuations among female MD patients (Liu et al., 2017; Aloufi et al., 2023).

The combination of ECochG and vestibular function examinations offers a more comprehensive assessment of the extent of target organ damage, helps understand residual functional status, and provides guidance for targeted rehabilitation treatments. However, several issues still need to be addressed. VEMPs can evaluate peripheral lesions, such as those in the labyrinth and vestibular neuropathy, and central pathway lesions in the brainstem. Additionally, VEMPs can be used to monitor disease progression and identify the active ear that requires treatment in patients with bilateral disease. Despite this potential, VEMPs have not yet been fully standardized as an emerging technology, and their value in neurological and otolaryngological diseases still needs to be better understood and improved through clinical practice to provide more objective evaluations. Furthermore, the sensitivity and specificity of vestibular function tests and ECochG in MD are not optimal, leading to a risk of missed diagnoses. Some of these tests are highly subjective, involve complicated procedures, and may cause patients discomfort, impacting the results and limiting their acceptance (Maes et al., 2011; Sandhu et al., 2012; Zhang et al., 2015). It remains to be seen whether the new staging method can effectively guide step-by-step medication, predict efficacy, and provide rehabilitation guidance for MD.

## 5 Conclusion

The integration of ECochG with vestibular function tests offers a more comprehensive and sensitive diagnostic framework for MD. The proposed staging system enhances diagnostic accuracy, particularly for early-stage disease, and provides valuable insights into the extent of cochlear and vestibular damage. This approach has significant clinical implications for guiding treatment strategies, monitoring disease progression, and improving patient outcomes. However, further research is needed to address the limitations and optimize the clinical application of these diagnostic tools.

## Data Availability

The original contributions presented in this study are included in this article/supplementary material, further inquiries can be directed to the corresponding author.
